# A narrative review of evidence to guide deprescribing among older adults

**DOI:** 10.1002/jgf2.464

**Published:** 2021-05-28

**Authors:** Kenya Ie, Shuichi Aoshima, Taku Yabuki, Steven M. Albert

**Affiliations:** ^1^ Division of General Internal Medicine Department of Internal Medicine St. Marianna University School of Medicine Kawasaki Japan; ^2^ Division of General Internal Medicine Department of Internal Medicine Kawasaki Municipal Tama Hospital Kawasaki Japan; ^3^ Department of Behavioral and Community Health Sciences University of Pittsburgh Graduate School of Public Health Pittsburgh PA USA; ^4^ Nakano Hospital Tochigi Japan; ^5^ Department of Internal Medicine Tochigi Medical Center Tochigi Japan

**Keywords:** deprescribing, intervention, Japanese healthcare system, older adults, polypharmacy

## Abstract

Potentially inappropriate prescription and polypharmacy are well‐known risk factors for morbidity and mortality among older adults. However, recent systematic reviews have failed to demonstrate the overall survival benefits of deprescribing. Thus, it is necessary to synthesize the current evidence to provide a practical direction for future research and clinical practice. This review summarizes the existing body of evidence regarding deprescribing to identify useful intervention elements. There is evidence that even simple interventions, such as direct deprescribing targeted at risky medications and explicit criteria‐based approaches, effectively reduce inappropriate prescribing. On the other hand, if the goal is to improve clinical outcomes such as hospitalization and emergency department visits, patient‐centered multimodal interventions such as a combination of medication review, multidisciplinary collaboration, and patient education are likely to be more effective. We also consider the opportunities and challenges for deprescribing within the Japanese healthcare system.

## Graphical abstract

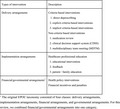

## 1 INTRODUCTION

The consequence of population aging and multimorbidity includes polypharmacy and adverse drug events.^1^ Polypharmacy may trigger drug interactions, adverse drug events, increased healthcare costs, and decreased adherence to medication.^2^ Adhering to current clinical practice guidelines in caring for an older person with multimorbidity may lead to polypharmacy,^3^ regardless of whether the prescription is to prevent life‐threatening events^4^, ^5^, ^6^, ^7^ or to alleviate symptoms. This is because most clinical guidelines are aimed at a single disease status.^3^

Many researchers have studied effective interventions for polypharmacy. However, recent systematic reviews regarding interventions to reduce polypharmacy have failed to demonstrate a benefit in clinical outcomes such as survival.^8^, ^9^, ^10^ Since potentially inappropriate prescribing and polypharmacy have the potential for adverse clinical outcomes, it is necessary to synthesize the current evidence to provide a practical direction for future research and clinical practice.

The prevalence of polypharmacy among older Japanese adults is relatively high,^11^, ^12^ and the number of drugs prescribed tends to increase until people are aged over 90 years, despite the evidence that the use of multiple drugs after the age of 80 poses a higher risk of adverse drug events.^13^ The Japanese healthcare system includes free access to healthcare facilities, which could lead to fragmentation of care without coordination (“polydoctoring”).^14^ The prevalence of polypharmacy is exceptionally high among older adults who visit multiple healthcare providers.^15^ The revised Japanese Geriatrics Society guidelines explicitly disclosed the risks associated with multidrug use and listed potentially inappropriate drugs.^16^ The risks of concomitant use of multiple drugs have also been covered in mass media, and awareness of the problem of polypharmacy is now widespread.^17^ Considering the impact of healthcare systems and cultures on medical practice, a narrative review outlining existing evidence with a focus on the Japanese context would help healthcare providers in Japan.

This review summarizes the existing body of evidence regarding polypharmacy interventions to elicit useful intervention elements and potential disadvantages. We also describe the prospects for which intervention methods could be practical or what approaches are needed within the Japanese healthcare system.

## 2 METHODS

We conducted a review of studies examining the effects of polypharmacy interventions among older adults. As overall reporting guidelines, we partially adopted PRISMA^18^ and the recommendation by Ferrari.^19^ SANRA^20^ was used for methodological rigor. A literature search of databases including PubMed, Embase, Google Scholar, Ichushi‐Web (Japanese medical literature), and J‐STAGE was conducted from inception to March 14, 2021. The researchers used “polypharmacy,” “intervention,” “deprescriptions,” “potentially inappropriate medications,” “medication reconciliation,” “clinical decision support systems,” “patient care teams,” “interdisciplinary communication,” “education,” and “feedback” as keywords. We cited individual randomized controlled trials (RCTs), nonrandomized interventional studies, observational studies, systematic reviews, narrative reviews, qualitative studies, and published gray literature as appropriate. Reference lists of the included studies were also used for further literature searches. We excluded studies that did not address any interventional aspects. Literature published in English and Japanese was adopted.

The Effective Practice and Organisation of Care (EPOC) taxonomy was adopted to classify the interventions into the elements of “delivery arrangements,” “implementation arrangements,” and “financial/governmental arrangements.”^21^ As this study was a narrative review of published sources, an ethical review was deemed unnecessary.

## 3 RESULTS

Polypharmacy interventions tend to be multifactorial. In a scoping review of intervention elements to reduce inappropriate prescribing by Lee et al., the included studies had an average of 2.5 intervention elements.^22^ More than 80% of the trials used more than one element.^22^ This review focuses on the major components of interventions and discusses them according to the EPOC taxonomy, classifying interventions as shown in Table 1. We focus especially on delivery arrangements and implementation arrangements to explore new perspectives on intervention approaches in clinical practice.

**TABLE 1:** Interventions for inappropriate polypharmacy based on EPOC taxonomy^21^

Types of intervention	Description
Delivery arrangements	Criteria‐based interventions Direct deprescribing Explicit criteria‐based interventions Implicit criteria‐based interventions ​ Non‐criteria‐based interventions Medication review Clinical decision support system (CDSS) Multidisciplinary team meeting (MDTM) ​
Implementation arrangements	Healthcare professional education Educational intervention Feedback Patient/family education ​
Financial/governmental arrangements^a^	Health policy interventions Financial incentives and penalties

^a^ The original EPOC taxonomy consisted of four classes: delivery arrangements, implementation arrangements, financial arrangements, and governmental arrangements. For this review, we combined financial/governmental arrangements into one category.

### 3.1 Delivery arrangements

Delivery arrangements are defined as “changes in how, when and where healthcare is organized and delivered, and who delivers healthcare,” called organizational interventions in the previous version of the EPOC taxonomy.^21^ We will summarize the interventions by dividing them into those that use specific criteria and those that do not.

#### 3.1.1 Criteria‐based interventions

These interventions for potentially inappropriate medications can be divided into three categories: (1) direct deprescribing, (2) explicit criteria‐based interventions, and (3) implicit criteria‐based interventions.

##### Direct deprescribing

The use of prophylactic medications such as cardiovascular drugs is common among older adults. Thus, healthcare providers need to continually evaluate on an individual basis whether continuing medication prescribing is still justified, in light of age‐related comorbidities and changes in frailty. Potential triggers for prophylactic drug deprescribing include current or anticipated adverse drug events, medication duplication and errors, and limited life expectancy.^23^ It is relatively straightforward to deprescribe prophylactic medications once the patient and the prescriber understand the risk–benefit of discontinuation. However, lack of understanding of the patient's perception of the medical condition and drug treatments can be a barrier to direct deprescribing.^24^, ^25^ Concerns about withdrawal symptoms, which are common with symptomatic medications, can also be a barrier to reduced prescribing.^26^ Although there is limited evidence to guide the reduction or cessation of individual drugs, several key articles can be utilized for patient‐centered communication and shared decision making (Table 2).

**TABLE 2:** Representative literature on the impact of deprescribing on specific drugs

Author, year	Target medications	Study design	Population	Interventions and main results
Boyé, 2017^114^	Fall Risk Increasing Drug (FRID)	RCT	Elderly patients who visited the emergency department due to falls (*N* = 612)	No significant difference in time to first fall with FRID discontinuation compared to usual care (HR 1.17; 95% CI 0.89–1.54)
Sheppard, 2020^115^	Antihypertensives	RCT	Older adults aged 80 years or older with two or more antihypertensives (*N* = 569)	A reduction of one antihypertensive drug does not significantly affect blood pressure control (RR, 0.98 [97.5% 1‐sided CI, 0.92 to ∞])
Kutner, 2015^116^	Statin	RCT	Patients with a life expectancy of 1 month to 1 year (*N* = 381)	No significant difference in death rate within 60 days between discontinuation and continuation groups (*p* = 0.36)
Sjöblom, 2008^117^	Blood glucose lowering medicine	Pre–post comparison	Nursing home residents with HbA1c <6% (*N* = 32, average age 84 years)	With the deprescribing of blood glucose lowering agents, there was less hypoglycemia and the average HbA1c was 5.8% after 3 months of intervention compared to 5.2% at baseline
Fraser, 2011^118^	Bisphosphonates	Meta‐analysis	Postmenopausal women and men older than 50 years (*N* = 1443)	Withdrawal after 5 years of continuation did not increase fracture risk. Nonspine fractures (RR 0.97; 95% CI 0.77–1.23)
Borlido, 2016^119^	Antipsychotics	RCT	Patients with schizophrenia taking multiple antipsychotic medications (*N* = 25)	No significant difference in the BPRS (Brief Psychiatric Rating Scale) between the group receiving continuous multidrug therapy and the group switching to monotherapy
Constantine, 2015^120^	Antipsychotics	RCT	Outpatients with clinically stable schizophrenia taking two antipsychotics (*N* = 104)	Symptoms worsened in the intervention group. Discontinuation was 13% in the usual care and 42% in the deprescribing group (*p* < 0.01)
Tannenbaum, 2014^68^	Benzodiazepines	Cluster RCT	Community‐dwelling elderly patients aged 65–95 years using BZD (*N* = 303)	Discontinuation of BZD after 6 months was 8.3 times more likely with educational interventions for BZD (risk explanation and tapering) (NNT 4.35)
Vicens, 2014^121^	Benzodiazepines	Cluster RCT	Patients taking BZD for at least 6 months（*N* = 532, median age 64 years）	Discontinuation of BZD after 12 months was about three times higher with educational interventions for physicians and gradual tapering (10–25% every 2–3 weeks)

Abbreviations: BZD, benzodiazepine; CI, confidence interval; HR, hazard ratio; RCT, randomized controlled trial; RR, risk ratio.

##### Explicit criteria‐based interventions

These are methods that use criteria (e.g., Beers Criteria,^27^ STOPP/START criteria^28^), or guidelines to screen for potentially inappropriate prescriptions to determine their inappropriateness. A systematic review by Hill‐Taylor et al. showed that interventions using STOPP/START reduced the rate of potentially inappropriate prescriptions.^29^ Kimura et al. reported a comparative study of 822 newly admitted patients before and after intervention using the STOPP/START criteria.^30^ In this study, 292 of 651 (44.9%) potentially inappropriate medications (PIMs) were changed or discontinued. A retrospective cohort study of 569 older adults admitted to a rehabilitation ward reported an association between a decrease in the Beers criteria PIMs and improvement in the Functional Independence Measure‐Motor at discharge.^31^ In Japan, the application of the Japan Geriatrics Society's Guidelines for Medical Treatment and its Safety in the Elderly 2015^16^ was reported to be a useful tool for deprescribing.^32^ However, the effect of explicit criteria intervention on clinically significant endpoints, such as death and rehospitalization, is unclear.

##### Implicit criteria‐based interventions

This approach determines the appropriateness of each drug, including indication, safety, efficacy, and manageability, for each patient. Scott et al. reported a five‐step deprescribing protocol comprising the following steps: (1) identify all the medications the patient is taking and the reasons for them; (2) assess the risk of adverse drug events in that individual; (3) evaluate the balance between current or potential future benefits and harms, (4) consider discontinuation preferentially from drugs with a higher risk than benefit and a lower likelihood of withdrawal symptoms or symptom recurrence, and (5) monitor carefully after the deprescribing.^33^ In an RCT comparing the deprescribing protocol with usual care in 95 patients aged ≥65 years, the number of drugs taken was reduced without significant adverse effects on survival or other clinical outcomes (The mean change in number of regular medicines at 12 months was −1.9 ± 4.1 in intervention group and +0.1 ± 3.5 in control group).^34^

#### 3.1.2 Non‐criteria‐based interventions

##### Medication review

Medication review is a comprehensive intervention, a structured evaluation of a patient's regimen to optimize medication use and improve health outcomes.^35^ In a 2020 review of inappropriate prescribing interventions for multimorbid older outpatients, 70% of studies included medication review, the most frequent of the 14 intervention elements.^22^

A 2016 Cochrane review of the effect of medication review on hospitalized patients showed a reduction in emergency department (ED) contacts (risk ratio (RR) 0.73 (95% confidence interval [CI] 0.52–1.03)), with a number needed to treat to prevent an ED contact of 37 for a low‐risk population and 12 for a high‐risk population (e.g., elderly patients, patients with multiple co‐medications) over one year.^36^ However, systematic reviews that did not limit the patient population to hospitalized patients did not show clinical benefit.^8^, ^9^, ^10^ This body of evidence suggests that the effects of medication reviews’ effects may be subtle unless targeted at high‐risk populations.

In an RCT by Ravn‐Nielsen et al., a composite of readmissions or ED visits were reduced in the multimodal intervention group utilizing medication review, combined with motivational interviewing and multidisciplinary team follow‐up, compared with the usual care among patients admitted to an acute care medical ward (HR, 0.77; 95% CI, 0.64–0.93).^37^ However, there was no significant difference in outcomes between the usual care and medication review alone groups. Similarly, interventions that combine patient interviews and patient education with medication review have been shown to reduce hospital visits and drug‐related hospitalizations^38^ and ED visits.^38^, ^39^ Patient‐centered multimodal interventions, such as a combination of medication review, multidisciplinary collaboration, and patient education, may be more effective than medication review alone.

##### Clinical decision support system

A clinical decision support system (CDSS) is designed to improve medical decisions with targeted clinical knowledge, patient information, and other health information.^40^ Evidence suggests that the use of CDSS was useful for reducing potential drug therapy problems in nursing homes^41^ and new PIMs in the elderly.^42^, ^43^ However, the clinical benefit of CDSS in health outcomes remains controversial. In a cluster RCT that combined CDSS and medication review in hospitalized patients, a per‐protocol analysis showed improvement in health‐related quality of life (QoL) measured by self‐rated global health (1: very poor; 5: very good) compared to usual care [mean: 3.14 (SD: 0.87) vs. 2.77 (0.94), *p* = 0.020].^44^ Another pilot RCT including 110 participants found that the use of CDSS, in addition to pharmacist intervention, considerably reduced re‐hospitalizations (RR, 0.65; 95% CI, 0.32–1.28) and ED visits (﻿RR, 0.62; 95% CI, 0.31–1.21) at 30 days.^45^ However, four other cluster RCTs found no effect of CDSS on clinical outcomes.^46^, ^47^, ^48^, ^49^ Considering that CDSS was combined with medication review by Bladh et al. and Elliott et al.,^44^, ^45^ we could conclude that CDSS alone does not sufficiently affect clinical outcomes.

##### Multidisciplinary team meeting (MDTM)

Several cluster RCTs have demonstrated that MDTMs improve Medication Appropriateness Index (MAI) scores^50^ and reduce psychotropic prescriptions,^51^ suggesting that problem‐solving‐oriented MDTMs may be effective in reducing PIMs. Recent studies from Japan also found that MDTMs reduce medication prescription during hospitalization,^52^ prescriptions for community‐dwelling adults with mental health problems,^53^ and prescriptions of hypnotics, anxiolytics, and antipsychotics.^54^ A nonrandomized controlled trial reported a nonsignificant mortality reduction with MDTM compared to the usual care group (6% vs. 15%, chi‐squared *p* = 0.07).^55^ However, MDTM alone has not been shown to improve clinical outcomes.

### 3.2 Implementation arrangements

Implementation arrangements for inappropriate polypharmacy involve a comprehensive, practice‐oriented approach to changing the culture of individual healthcare professionals and organizations.^21^ They include education and feedback for healthcare professionals and patient education.

#### 3.2.1 Interventions for healthcare professionals

##### Educational interventions

Educational interventions for medical professionals are often targeted at physicians. They range from simple (e.g., the use of explicit criteria such as the STOPP/START) to a more comprehensive set of educational sessions (e.g., the pharmacokinetics of the elderly, comprehensive geriatric assessment, basic knowledge of polypharmacy).

Pre–post comparison studies examining the effects of explicit criteria‐based physician education have reported a decrease in the number of medications and PIMs and improved MAI scores.^56^, ^57^ However, the effects of educational interventions using an individualized implicit approach have been inconsistent. For example, a cluster RCT that educated clinicians regarding comprehensive geriatric assessment, the pharmacokinetics of the elderly, and PIMs through e‐learning did not reduce PIMs.^58^ Another cluster RCT that implemented a 10‐h educational program and telephone consultation service targeted at physicians revealed a significant PIM reduction and drug duplication in the intervention group.^59^ Neither study showed improvements in meaningful clinical endpoints. However, a cluster RCT conducted in Australian general practices revealed a significant fall reduction [adjusted odds ratio (OR), 0.61; 95% CI, 0.41–0.91] by physician education in combination with medication review and financial incentives.^60^

In clinical practice, pharmacists play an important role in medication safety. Studies examining the effect of educational interventions targeted at pharmacists report improvement in the rate of adverse drug reaction reporting.^61^, ^62^, ^63^ However, evidence is scarce regarding the effect of pharmacist education on clinical endpoints.^64^

##### Feedback

Feedback interventions including prescription review and prescriber feedback have been shown to be effective. These include sending recommendations by letter^65^ or fax^66^ and have been shown to be effective in reducing the number of drugs and PIMs. In addition to reducing PIMs, prescriber feedback affects physicians’ overall prescribing behavior.^67^ Prescription monitoring, physician feedback, and improving transparency may be useful approaches for reducing inappropriate polypharmacy.

#### 3.2.2 Patient education

In addition to health professional education, patient education plays a crucial role in deprescribing. A Canadian cluster RCT that examined the effect of pharmacist‐led patient education on patients with chronic benzodiazepine use showed a significant reduction in the benzodiazepine prescription, with a discontinuation rate of 27% in the intervention group and 5% in the control group.^68^ In a similar cluster RCT of older patients taking PIMs, explaining drug information to patients using educational pamphlets significantly reduced inappropriate prescribing after six months.^69^

An Australian report prioritized elements of practical interventions. “Pharmacist‐led medication reconciliation for new residents,” “facility‐level drug audits and feedback to healthcare providers and staff,” and “prescription scripting to support physician–patient discussions” were deemed to be high priorities for deprescribing among facility residents.^70^

### 3.3 Financial/governmental arrangements

Policy and economic incentives potentially influence prescribing behavior. In the United States, Medicare plays an essential role in reducing polypharmacy. The Centers for Medicare and Medicaid Services also implemented a program to improve the quality of care for older adults with dementia; it included educational programs and prescription monitoring and penalties for facilities with high numbers of PIMs.^71^ As a result, the frequency of psychotropic medication prescriptions among institutionalized patients in the United States decreased by one‐third between 2011 and 2016.^72^ Project SYMPATHY in the E.U. is an initiative against polypharmacy and drug nonadherence. The project includes medication reviews, prescription adjustments, and the promotion of PIM detection tools (mainly STOPP/START criteria), and is planned to examine the effectiveness of interventions in the future.^71^ In Australia, pharmacists have been required to conduct medication reviews for institutionalized patients since 1998. There is evidence that such pharmacist‐led mandatory medication review is useful for detecting prescribing‐related problems.^71^

The effects of different types of policy approaches have been studied. Prescription monitoring, driver's license restrictions for benzodiazepine users, changes in prescription monitoring regulatory classifications, public awareness campaigns, discontinuation of payments by public insurance, and financial incentives for withholding benzodiazepine were compared. Prescription monitoring was the most effective in reducing PIMs.^73^

Unintended effects of policy‐level interventions have also been evident: Alprazolam prescription regulation led to a decrease in prescriptions, accompanied by an increase in street drug prices^74^ and deaths from overdoses of other benzodiazepines.^75^ Benzodiazepine coverage restrictions in U.S. Medicare increased Z drug prescriptions and patient co‐payments.^76^, ^77^ Furthermore, it has been pointed out that vulnerable populations are more likely to be adversely affected by policy‐level interventions.^78^

In Japan, the revision of reimbursement from 2012 to 2016 confirmed a decrease in the rate of inappropriate psychotropics prescribing.^79^ In 2016, the Japanese government endorsed a reimbursement system for deprescribing (Yakuzai‐Sougou‐Hyouka‐Chousei‐Kasan for inpatients and Yakuzai‐Sougou‐Hyouka‐Chousei‐Kanriryou for outpatients) when healthcare facilities reduce two or more medications among those prescribed more than six medications. The Ministry of Health, Labor and Welfare published “Guidelines for the Appropriate Use of Medicines by the Elderly (2018)” to further promote adequate prescribing among older adults.^80^ Nevertheless, a 2019 domestic survey revealed that 65% of hospitals and 70% of clinics had never claimed reimbursement for deprescribing in the past year.^81^ This result may indicate that there are barriers for deprescribing such as the shorter hospital stay and “polydoctoring,” or that the reimbursement system for deprescribing has not been widely recognized. Further dissemination of the policy and verification of effectiveness, as well as removing these barriers, are required.

### 3.4 Disadvantages of interventions

Few studies have scientifically examined the disadvantages of deprescribing. However, in clinical practice, healthcare providers often face challenges in reducing inappropriate medications due to concerns about symptom recurrence, drug withdrawal symptoms, and relationship deterioration among patients and healthcare providers.

#### 3.4.1 Symptom recurrence and disease development related to deprescribing

Previous studies have examined the risk of symptom recurrence and disease development due to deprescribing interventions in several drug categories.

##### Antihypertensive medications

Deprescribing of antihypertensive medications is considered when adverse drug events, such as dizziness, fainting, falls, and fall‐related injuries, are possible. In RCTs that examined the effects of reducing antihypertensive medications in older adults, blood pressure increased by about 7–15 mmHg immediately after discontinuation, then gradually returned to baseline within nine months.^82^, ^83^ Patients with optimal blood pressure control may benefit from knowing that deprescribing may raise their blood pressure temporarily, but in time it may settle down.

##### Proton pump inhibitors

Dose reduction or cessation of proton pump inhibitors (PPIs) have been associated with symptom recurrence. A 2017 Cochrane review compared gastrointestinal symptoms between dose reduction and continuation in patients with long‐term PPI use.^84^ The study found that gastrointestinal symptoms could occur within two weeks of discontinuation. Tapering or on‐demand deprescribing was also associated with symptom recurrence. In a Swedish RCT that examined PPI tapering in long‐term users, only 27% of patients in the intervention arm could discontinue PPIs.^85^ When deprescribing symptomatic medications, clinicians should explain to patients that symptom recurrence may occur and that they can restart the medication at any time if necessary. A common understanding is essential before initiating deprescribing.

#### 3.4.2 Withdrawal symptoms with deprescribing

Certain drugs can cause withdrawal symptoms upon discontinuation. The most famous drug class includes benzodiazepines, but several other drugs can also cause withdrawal symptoms^86^ (Table 3). Attention should be paid when reducing these medications in patients who have been taking the drug for a certain amount of time.

**TABLE 3:** Drugs likely to cause withdrawal symptoms [adopted from Bangert et al.^86^]

Drug class	Withdrawal symptoms related to cessation or dose reduction
Opioids	Anxiety, irritability, agitation, sweating, tremors, chills, lachrymal secretion, nasal discharge, loss of appetite, nausea, vomiting, convulsions, mydriasis, tachycardia, hypertension, increased pain, craving for drugs
Benzodiazepines	Risks are particularly high for short‐acting benzodiazepines *Physical effects:* fatigue, weakness, muscle tension, cramps, pain, sweating, tremors, trembling, tachycardia, hypertension, anorexia, symptomatic seizures *Psychological effects:* anxiety, agitation, restlessness, depression, emotional instability, difficulty concentrating, delirium, delusions, hallucinations, loss of sense of reality, insomnia *Sensory effects:* auditory hypersensitivity, photophobia, paresthesia, tinnitus, blurred vision
Barbiturates	*Physical/autonomic effects:* weakness, sweating, nausea, vomiting, fatigue, headache, dry mouth, fever *Psychological effects:* insomnia, anxiety, insecurity, nervousness, depression, hallucinations, delirium *Neurological effects:* tremor, myoclonus, convulsions, seizures *Severe withdrawal symptoms:* recurrent grand mal seizures and delirium, death
Baclofen	Psychosis, auditory and visual hallucinations, mood disorders, agitation, insomnia, confusion, delirium, tachycardia, sweating, rhabdomyolysis and muscle cramps, seizure/status epilepticus, subarachnoid baclofen withdrawal symptoms—potentially fatal
Beta‐blockers	*Tachycardia:* sinus tachycardia, supraventricular or ventricular tachycardia Nervousness, anxiety, agitation, headache, sweating, tremors, nausea *Hypertensive crisis* *Serious complications:* angina pectoris, myocardial infarction, sudden death
Corticosteroids	Severe fatigue and malaise, low blood pressure, tachycardia, muscle pain, joint pain, dizziness, mood disorder, depression, loss of appetite, nausea, vomiting, diarrhea *Severe withdrawal symptoms:* fever, shock, and death
Gabapentin and pregabalin	Anxiety, restlessness, agitation, tachycardia, catatonia, seizures, sweating, hypertension, diarrhea, tremor, increased spasticity, auditory hallucinations, self‐injurious behavior, suicidal tendencies, delirium, confusion
Dopamine agonists	*Psychiatric effects:* anxiety, panic attacks, depression, suicidal thoughts, agitation, irritability, confusion *Autonomic/gastrointestinal effects:* malaise, nausea, vomiting, orthostatic hypotension, sweating, flushing *Sensory effects:* diffuse pain, restless legs syndrome
Antidepressants	*Flu‐like symptoms:* headache, body aches, lethargy, fatigue *Sleep disturbances:* insomnia, nightmares, vivid dreams *Sensory disturbances:* tingling, dysesthesia, burning sensation *Psychological disorders:* unstable emotions, anxiety, restlessness, mania, cognitive impairment *Gastrointestinal disorders:* nausea, diarrhea, dry mouth *Equilibrium disorders:* ataxia, dizziness, lightheadedness, vertigo

#### 3.4.3 Communication errors with patients

Patients with polypharmacy may feel that they have too many medications and be willing to discontinue them if they know that their physician can resume them as necessary.^87^, ^88^ Some patients are reluctant to reduce their medications if they expect them to have preventive effects in the future.^89^ Moreover, if patients are anxious about discontinuing a drug, they may be more likely to experience side effects when the drug is reduced or discontinued (nocebo effect).

Healthcare providers should understand such feelings and expectations about the target medication. One study that evaluated opioid deprescribing in patients with chronic pain found that nocebo effects could be minimized by thoroughly educating patients about the benefits of medication reduction and reducing opioids more slowly than the standard duration.^90^ The keys to successful intervention are to provide clear guidance on discontinuation, reduce patient anxiety, and create an individualized drug reduction protocol. Clinicians need to balance these keys with the amount of time and effort required.

#### 3.4.4 Communication errors among healthcare professionals

Clinicians’ attitudes toward prescription‐related problems vary. In addition to physician factors (e.g., beliefs, attitudes, knowledge, skills, and behaviors), many external factors (e.g., work environment, healthcare system, culture) influence their clinical decision.^91^ Clinicians’ inertia is a characteristic that makes them more likely to continue potentially inappropriate prescriptions. It was reported that general practitioners were more likely to continue prescribing, because of uncertainty and lack of information.^92^, ^93^, ^94^ They are also less likely to discontinue a drug prescribed by another specialist,^95^, ^96^, ^97^ probably due to concern about deterioration in the physician–physician relationship.^98^

## 4 DISCUSSION

### 4.1 Summary

Evidence on hard endpoints such as mortality, hospitalization, and falls is scarce and further research is needed. Improving process indicators, such as the number of prescribed medications and PIMs, can be achieved with any approach in the EPOC taxonomy. CDSS, MDTM, and educational interventions are useful, but direct deprescribing targeted at Fall Risk Increasing Drugs and explicit criteria‐based approaches can also effectively reduce PIMs. But if the goal is to improve clinical outcomes such as death, hospitalization, falls, and QoL, patient‐centered multimodal interventions such as a combination of medication review, multidisciplinary collaboration, and patient education are likely to be more effective. Table 4 summarizes the evidence of RCTs on the effect of deprescribing based on EPOC taxonomy.^21^

**TABLE 4:** Evidence summary of randomized controlled trials in the effect of deprescribing based on EPOC taxonomy^23^

Deprescribing element	Effect on medication use	Effect on clinical outcomes
**Delivery arrangements**		
*Criteria‐based interventions*		
1. Direct deprescribing	*****Positive evidence***** ‐Two third of patients in deprescribing preventive cardiovascular medication group quit the medication after 2 years^122^ ‐Significant reductions in long‐term benzodiazepine use in patients without severe comorbidity^121^ ***Negative evidence*** ‐Percentage of users with three or more FRIDs did not change^114^	***Positive evidence*** ‐Reducing hypertensive medications did not alter blood pressure control^115^ ‐Stopping statin is safe and may be associated with benefits including improved QoL, use of fewer nonstatin medications, and a reduction in medication costs^116^ ‐No significant difference in fracture risk between those continued bisphosphonate and those discontinued^118^ ***Negative evidence*** ‐Reducing FRIDs had no effect on fall^114^ ‐Participants who switched to antipsychotic monotherapy experienced greater increases in symptoms than stay patients^120^
2. Explicit criteria‐based interventions	***Positive evidence*** ‐STOPPFrail‐guided deprescribing significantly reduced polypharmacy and medication costs in frail older people^123^ ‐Greater discontinuation of inappropriate prescriptions^69^ ‐The intervention with the STOPP/START criteria reduced the number of potential prescribing omissions in the elderly with advanced chronic kidney disease^124^ ‐The use of STOPP/START criteria reduced number of drugs prescribed and drug costs^125^ ‐The use of STOPP/START criteria reduced PIMs prescription^29^	***Positive evidence*** The use of STOPP/START criteria reduced number of falls^125^ ***Negative evidence*** ‐No detectable impact on medication adherence or health‐related quality of life^124^ ‐The use of the criteria showed no evidence of improvements in quality of life or mortality^29^
3. Implicit criteria‐based interventions	***Positive evidence*** ‐The mean change in number of medicines at 12 months was −1.9 in intervention group participants and +0.1 in control group participants^34^ ***Negative evidence*** ‐Patient‐centered deprescribing procedure is effective immediately after the intervention, but not after 6 and 12 months^99^	***Negative evidence*** ‐The deprescribing protocol had no effect on clinical outcomes including survival^34^
*Non‐criteria‐based interventions*		
1. Medication review	***Positive evidence*** ‐Reduction of medication discrepancies (Meta‐analyses of RCTs)^126^, ^127^, ^128^ ***Negative evidence*** ‐Clinically important medication errors were not reduced by a medication review, low‐literacy adherence aids, and individualized telephone follow‐up^129^ ‐The effect of medication reconciliation on medication discrepancies and adverse drug events was not significant^130^	***Positive evidence*** ‐Medication review in hospitalized adult patients may reduce emergency department contacts: risk ratio 0.73 (95% CI 0.52–1.03) (Meta‐analysis of RCTs)^36^ ‐ED visits and readmissions were reduced by a multimodal intervention utilizing medication review, motivational interviewing, and multidisciplinary team follow‐up^37^ ‐Interventions that combine patient interviews and patient education with medication review reduced hospital visits, drug‐related hospitalizations,^38^ and ED visits^38^, ^39^ ‐Medication reconciliation, a patient‐specific pharmaceutical care plan, discharge counseling, and postdischarge phone calls reduced a composite of readmission or ED visit^131^ ***Negative evidence*** ‐No effect on mortality or hospital readmissions (meta‐analysis of RCTs)^36^ ‐Little or no difference on unplanned rehospitalization when reported alone (meta‐analysis of RCTs)^128^ ‐No effect on all‐cause mortality(Meta‐analysis of RCTs)^8^ ‐No effect was found on mortality, hospital admissions/healthcare use, the number of patients falling, physical and cognitive functioning(Meta‐analysis of RCTs)^10^
2. Clinical decision support system	***Positive evidence*** ‐Implementing CDSS reduced potential drug therapy problems^41^ and PIMs prescription^42^, ^43^ ‐Significantly more appropriate drug orders^132^ ***Negative evidence*** ‐No effects on the overall number of adverse drug events^133^	***Positive evidence*** ‐CDSS with medication review improved HR‐QoL^44^ ‐In a small pilot RCT with 110 participants, CDSS considerably reduced re‐hospitalizations and ED visits^45^ ***Negative evidence*** ‐No change in hospitalization, emergency department use, and medication regimen complexity^49^ ‐No effect on clinical outcomes^46^, ^47^, ^48^
3. Multidisciplinary team meeting	***Positive evidence*** ‐Improve MAI score^50^ ‐Reduction in psychotropics prescription^51^ ‐Fewer medications among patients with psychiatric disorders^53^ ‐Reducing hypnotics, anxiolytics, and antipsychotics^54^	*Evidence is still emerging*
**Implementation arrangements**		
*Healthcare professional education*		
1. Educational intervention	***Positive evidence*** ‐Physician education utilizing explicit criteria or PIMs reduced number of medications, PIMs prescription, and MAI score^60^, ^134^ ‐Interactive training sessions for nursing staff can reduce the use of harmful medications^135^ ‐Educational intervention on drug use improves the use of inappropriate drugs, use of antipsychotics, and drug duplications in their residents^59^	***Positive evidence*** ‐Activating learning methods directed at nurses can maintain HRQoL and reduce hospitalization^135^ ‐Physician education in combination with medication review and financial incentives reduced fall (aOR, 0.61; 95% CI, 0.41–0.91)^60^ ‐Educational intervention on drug use may also improve the risk of delirium and falls, and reduce the use of healthcare resources^59^ ***Negative evidence*** ‐No effect of the tailored program on the combined primary outcome^59^, ^134^ ‐Generic education was not effective^102^
2. Feedback	***Positive evidence*** ‐Direct feedback to GPs have shown to be effective in reducing the number of drugs and Improve MAI score^136^, ^137^, ^138^ ***Negative evidence*** ‐No changes were seen in PIMs and medication reviews in elderly patients after an educational intervention with feedback in primary care^139^	***Negative evidence*** ‐No mortality change^138^ ‐No changes were seen in acute health care consumption in elderly patients after an educational intervention in primary care^139^
Patient / family education	***Positive evidence*** ‐Pharmacist‐led patient education showed a significant reduction in benzodiazepine prescription^68^ ‐Explaining drug information to patients using’ educational pamphlets significantly reduced inappropriate prescribing^69^ *****Negative evidence***** ‐Doctor–patient dialogue and discussing the patient agenda did not lead to a reduction of medication intake.^140^	***Negative evidence*** ‐The doctor–patient dialogue and discussing the patient's agenda and personal needs did not alter health‐related quality of life^140^ ‐Hospitalization not signifcant^68^, ^69^ ‐No mortality change^69^

Abbreviations: aOR, adjusted odds ratio; CDSS, clinical decision support system; CI, confidence interval; ED, emergency department; FRID, Fall Risk Increasing Drug; GP, general practitioner; HR‐QoL, health‐related quality of life; MAI, Medication Appropriateness Index; PIM, potentially inappropriate medication; QoL, quality of life; RCT, randomized controlled trial; START, Screening Tool to Alert to Right Treatment; STOPP, Screening Tool of Older Persons’ Prescriptions.

### 4.2 Why are the clinical effects of polypharmacy interventions difficult to prove?

Many polypharmacy interventions reduce the number of prescribed medications and PIMs but do not improve clinical outcomes. We hypothesized that there are two reasons for this.

First, both morbidity and polypharmacy interventions in older adults are multifactorial. The benefit of prophylactic medications for chronic diseases in older adults is relatively small compared to that in the younger population. Furthermore, the impact of potentially inappropriate prescribing may not be significant among the multiple factors associated with mortality and morbidity in the elderly. In addition, deprescribing is not always targeted at drugs with potentially significant harm. If the effect size of uniform polypharmacy intervention is not substantial, future studies need to clarify what kind of deprescribing approach is most likely to offer benefits for particular patient populations.

Second, the indirect effects on usual care may have dampened the effects of polypharmacy interventions. For instance, short‐term indirect effects include contamination bias associated with intervention implementation. In a cluster RCT in Switzerland^99^ and a pre‐ and postintervention study in Japan,^100^ deprescribing interventions were associated with a decrease in the number of medications used in the usual care group. It is possible that the implementation of polypharmacy interventions may have a desirable impact on culture and usual care at their institutions. A medium‐ to long‐term indirect impact is that awareness of polypharmacy may have improved the quality of usual care over the past few decades. Just as the Surviving sepsis campaign has contributed to the reduction of sepsis mortality by improving the quality of care for sepsis over time,^101^ deprescribing interventions may contribute to quality improvement in the standard of care.

### 4.3 What interventions are recommended?

A 2016 meta‐analysis found no survival benefit for deprescribing.^102^ However, subgroup analysis found that patient‐specific interventions reduced mortality (OR, 0.62 [0.43–0.88]), while generalized educational programs did not reduce mortality. Medication review may improve clinical outcomes when combined with other elements such as motivational interviewing, patient education, and multidisciplinary collaboration.^37^, ^38^, ^39^ Rather than avoiding adverse drug events by reducing the number of medications themselves, changes in patients’ and providers’ perceptions through multifaceted interventions that involve patients may be associated with improved clinical outcomes. In particular, it has been suggested that patient activation and improved self‐control over drug therapy improve clinical outcomes.^103^ A patient‐centered, shared decision‐making model of deprescribing is a possible model that has been proposed recently.^104^

Problem‐solving‐oriented MDTMs are effective in reducing potentially inappropriate prescribing and healthcare costs but do not improve clinical outcomes. However, indirect benefits of MDTMs, such as nurturing trust and organizational culture in the workplace, could be expected in facilities that address polypharmacy.^22^ The American Geriatrics Society has proposed five critical elements for effective multidisciplinary collaboration: (1) shared goals and objectives, (2) clarification of roles and responsibilities, (3) appropriate contributions of team members, (4) cooperation and coordination in activities, and (5) fostering mutual trust through ongoing relationships.^105^

### 4.4 Problems to be solved in the Japanese context

Japan has had universal medical coverage since 1961, and copayment is inexpensive by global standards.^106^ Access to health care is good because patients can visit medical institutions without paying much attention to costs. However, this accessibility tends to lead to excessive medical care.^107^ The rate of inappropriate prescribing was higher among those fully exempted from public payment for medical services.^108^ Recently, public education via mass media has become widespread. This leads to public opinion that polypharmacy is a significant public health concern.^17^ A resulting problem is the psychological avoidance of problems related to polypharmacy.^109^ This is because oversimplified criticism of polypharmacy, which ignores each case's context, has sometimes been disseminated via mass media and medical professionals. However, it has been accepted that there are “appropriate polypharmacy” and “problematic polypharmacy” and that polypharmacy and the use of PIMs are not uniformly harmful.^110^ Another disadvantage of free access is polydoctoring, that is, patients seeking care from multiple providers. Since polydoctoring is a known risk factor for polypharmacy,^15^ it is necessary to establish an environment in the Japanese healthcare system that encourages people to utilize primary care physicians as the point of contact for care, as well as better multidisciplinary cooperation. As stated before, the recent reimbursement system for deprescribing may not been widely recognized. Adequate public awareness campaign of the system, further incentives for deprescribing, and the implementation of hospital dashboard including the rate of acquisition of the deprescribing reimbursement would be expected to promote dissemination of the policy. Recently, pharmacists in clinical practice have become actively involved in pharmacotherapy through multidisciplinary collaboration.^111^, ^112^ Nevertheless, a lack of trust and communication among pharmacists and prescribers can be a barrier and should be addressed.^113^

### 4.5 Future implications

As mentioned, reducing the number of prescribed medications and PIMs does not directly lead to improved clinical outcomes. Thus, it is necessary to recognize that the number of drugs is no more than an intermediate factor. Nevertheless, using the number of drugs as a surrogate endpoint, patient‐centered multifaceted intervention may improve clinical outcomes by changing patients’ and providers’ perspectives concerning their health. Future studies are needed to clarify the essential elements in improving clinical outcomes. Several elements, such as education on polypharmacy prevention, prescribing restrictions, inappropriate prescribing alerts, revision of guidelines focusing on polypharmacy prevention, and implementation of local formularies, may be vital. We have summarized the challenges for future polypharmacy interventions from the Japanese primary care perspective in Table 5.

**TABLE 5:** Challenges for future polypharmacy interventions from Japanese primary care perspectives

Areas of inquiry	Descripion of agendas
What are the useful combinations of interventions?	There is no single intervention that can be expected to improve clinical outcomes, and patient‐centered multifaceted interventions combined with medication review may be effective. It is necessary to examine what mechanisms work behind such relationships and which factors play a crucial role in clinical effectiveness.
Fostering a culture of preventing and reducing inappropriate polypharmacy in medical practice	Trust and “culture” between professions is a prerequisite for a useful medication review. What is necessary to develop such a culture? What process should be used to do so at each medical facility?
What are the subgroups for which interventions are most effective?	Assuming that the effect size of uniform polypharmacy intervention is not substantial, future studies need to clarify what kind of deprescribing approach is most likely to offer benefit for particular patient populations.
Studies using patient‐centered outcomes	It is necessary to examine the effect of interventions on patient‐centered outcomes such as health‐related quality of life. Likewise, we recommend evaluating the quality of care from multiple perspectives, including QoL and patient experience.
Research from the perspective of medical practice in Japan	It is worth examining whether concepts proposed and interventions proven in Europe and the United States are also useful in Japan's cultural context and the healthcare system. Evidence in the Japanese context, especially from qualitative and mixed methods studies, is needed.
Examining the effects of policy interventions	The impact of the 2016 implementation of the fee for deprescribing on physicians’ prescribing behaviors, patient outcomes, and healthcare costs should be examined.

Cross‐disciplinary approaches, such as behavioral economics, may become more critical in developing practical approaches to inappropriate polypharmacy. This is because patients’ health care‐seeking behavior and doctors’ clinical decisions are often not based merely on scientific evidence. It is further necessary to educate the public so that polypharmacy is viewed as an opportunity to seek better medical care.

## CONFLICT OF INTEREST

Authors declare no conflict of interests for this article.
